# *Synhelminthosporium* gen. et sp. nov. and Two New Species of *Helminthosporium* (Massarinaceae, Pleosporales) from Sichuan Province, China

**DOI:** 10.3390/jof8070712

**Published:** 2022-07-05

**Authors:** Yanpeng Chen, Wenhui Tian, Yaobin Guo, Hugo Madrid, Sajeewa S. N. Maharachchikumbura

**Affiliations:** 1 School of Life Science and Technology, Center for Informational Biology, University of Electronic Science and Technology of China, Chengdu 611731, China; yanpengch@std.uestc.edu.cn (Y.C.); wenhuitian@std.uestc.edu.cn (W.T.); imguoyaobin@163.com (Y.G.); 2 Departamento de Tecnología Médica, Facultad de Ciencias de la Salud, Universidad de Tarapacá, Sede Iquique, Av. Luis Emilio Recabarren 2477, Iquique 1100000, Chile; hugo.madrid@gmail.com

**Keywords:** Ascomycota, Dothideomycetes, fungal taxonomy, morphology, multi-locus, phylogeny

## Abstract

*Helminthosporium* is a polyphyletic genus in Massarinaceae (Pleosporales). Species of *Helminthosporium* are characterized by having septate and erect conidiophores, acro-pleurogenous and distoseptate conidia with a ring-shaped scar at the base. During a survey of fungal diversity in Sichuan Province, China, six *Helminthosporium*-like isolates were collected from dead branches of unknown trees. Five barcodes, including ITS (ITS1-5.8S-ITS2), SSU, LSU, *TEF1*, and *RPB2* were amplified and sequenced. Morphological examination and multi-locus phylogenetic analyses revealed two new *Helminthosporium* species (*H. chengduense* sp. nov., and *H. chinense* sp. nov.), a new genus (*Synhelminthosporium* gen. nov.) with a type species *Synhelminthosporium synnematoferum* sp. nov., and two known species (*Helminthosporium submersum* and *H. velutinum*) within Massarinaceae. The new genus *Synhelminthosporium* differs from the phylogenetically closest genus *Helminthosporium* by producing synnematous conidiophores. This work expands our understanding of the diversity of *Helminthosporium*-like taxa in Sichuan Province, China.

## 1. Introduction

Fungi consist of a highly diverse lineage of eukaryotes with a huge estimated number of between 2.2 and 3.8 million species [1]. Investigating fungal diversity is vital in Assembling the Fungal Tree Of Life (AFToL) [2], which significantly enhances our understanding of the history of life and also strengthens our ability to explore and use fungal resources [3].

*Helminthosporium* is an old, species-rich genus erected by Link in 1809 [4]. In addition to *Helminthosporium*, ten other genera are accepted in the family Massarinaceae (Pleosporales, Dothideomycetes), i.e., *Byssothecium*, *Haplohelminthosporium, Helminthosporiella*, *Massarina*, *Mirohelminthosporium*, *Pseudodidymosphaeria*, *Pseudosplanchnonema*, *Semifissispora*, *Stagonospora*, and *Suttonomyces* [5,6]. Based on multi-locus phylogenetic analysis, Konta et al. [7] confirmed that *Helminthosporium* is polyphyletic, where members were mixed with other taxa of *Byssothecium*, *Helminthosporiella*, and *Pseudosplanchnonema*. Most *Helminthosporium* species are saprobes feeding on dead or decaying woods [7,8]. However, one species, *H. solani* is an economically important pathogen causing silver scurf disease in potatoes worldwide [9,10]. *Helminthosporium* species are commonly collected from leaves and decaying wood in terrestrial habitats [7,8,11] and rarely reported in freshwater habitats [12].

The genus *Helminthosporium*, typified by *H. velutinum*, is characterized by producing macronematous, cylindrical, rather straight, septate, erect conidiophores with tretic conidiogenous cells and clavate or obclavate, distoseptate conidia with a flat, ringed pore at the base [8,13]. Conidia are produced mainly laterally from tretic conidiogenous cells and the production of a terminal conidium usually determines the end of conidiophore growth. Most *Helminthosporium* species were introduced based on their asexual morph and only six species, viz., *H. tiliae*, *H. microsorum*, *H. oligosporum*, *H. massarinum*, *H. quercicola*, and *H. quercinum*, were characterized based on both morphs [8,11]. *Splanchnonema kalakadense* was described as the sexual morph of *H. velutinum*, but this was only based on pure culture without sequence data [14]. Tanaka et al. [11] first connected the *Massarina*-like sexual morph and asexual morph of *H. massarinum*, which was confirmed based on pure culture and sequence data. Voglmayr and Jaklitsch [8] experimentally confirmed three *Splanchnonema*-like sexual morphs of *Helminthosporium* species based on pure culture, sequence data, and herbarium studies, which extends the knowledge of sexual morphs of *Helminthosporium*.

Sichuan Province, located in southwestern China, along the Yangtze River, has enormous fungal diversity [15,16,17,18]. We regularly conduct fungal diversity surveys in Sichuan Province. During the study of preliminary morphological examination and BLASTn analysis of ITS sequences (the ribosomal internal transcribed spacer), a total of six *Helminthosporium*-like isolates were obtained from July to September 2021. Based on the multi-locus phylogenetic analysis and morphological examination, two known *Helminthosporium* species including a new habitat record, two new *Helminthosporium* species, and a new genus *Synhelminthosporium* with the type species, *S. synnematoferum* sp. nov. are introduced. This study broadens our understanding of the diversity of *Helminthosporium*-like taxa.

## 2. Materials and Methods

### 2.1. Sample Collection, Isolation, and Morphological Examination

A survey of the diversity of ascomycetous fungi in Sichuan Province, China, was conducted between July and September 2021. Dead branches were collected from three locations in Sichuan Province (Yunqiao Wetland, Chengdu City; Baiyungou, Chongzhou City; Huilonggou, Pengzhou City). The specimens were taken to the laboratory in paper envelopes for examination. The morphological observation was consistently carried out from material on natural substrates. Tiny pieces of mycelium were mounted in a drop of sterilized water using syringe needles. Microscopic characters were observed and recorded using a Nikon SMZ800N stereo microscope equipped with a Nikon DS-Fi3 microscope camera and a Nikon ECLIPSE Ni-U microscope fitted with a Nikon DS-Ri2 microscope camera, respectively. Measurements were conducted using the Nikon NIS-Elements Documentation Imaging Software Version 5.21.00. All photos were processed using Adobe Photoshop software version 22.0. Isolates were obtained by picking up pieces of mycelium into sterilized water, spreading the suspension onto the surface of potato dextrose agar (PDA) plates, and incubated for 24 h at 25 °C. Germinated conidia were individually transferred to PDA plates and incubated under the dark at 25 °C. Culture characteristics were examined and recorded after one week and later at regular intervals.

The specimens were deposited in the Herbarium of Cryptogams Kunming Institute of Botany Academia Sinica (HKAS), Kunming, China, or the Herbarium of University of Electronic Science and Technology (HUEST), Chengdu, China. The living cultures were deposited in the China General Microbiological Culture Collection Center (CGMCC) in Beijing, China, and the University of Electronic Science and Technology Culture Collection (UESTCC) in Chengdu, China.

### 2.2. DNA Extraction, PCR Amplification, and Sequencing

Fungal genomic DNA was extracted from mycelia using the Trelief^TM^ Plant Genomic DNA extraction Kit (TSINGKE Biotech, Shanghai, China) according to the manufacturer protocol. The DNA was stored at −20 °C for long-term storage. Five barcodes including the nuclear ribosomal internal transcribed spacer region (ITS: ITS1-5.8S-ITS2), the partial nuclear ribosomal small subunit rRNA gene (SSU), the partial nuclear ribosomal large subunit rRNA gene (LSU), the partial translation elongation factor 1-alpha gene (*TEF1*) and the partial second-largest subunit of RNA polymerase II gene (*RPB2*) were amplified by polymerase chain reaction (PCR). The corresponding primer pairs and PCR processes are listed in Table 1. The final PCR reaction volume was 25 µL containing 2 µL of DNA template, 1 µL each of the forward and reverse primer (10 µM), 8.5 µL of double-distilled water (ddH_2_O), and 12.5 µL of 2 × Flash PCR MasterMix (mixture of DNA Polymerase, dNTPs, Mg^2+^ and optimized buffer; CoWin Biosciences, Taizhou, China). The PCR products were visualized in 1% agarose gel electrophoresis. Sanger sequencing was conducted by Tsingke Biological Technology (Beijing, China).

### 2.3. Phylogenetic Analyses

According to the corresponding Sanger sequencing chromatograms, misleading data from the ends of raw sequencing fragments were manually trimmed and assembled into consensus sequences using SeqMan Pro version 7.1.0 (DNASTAR, Inc. Madison, WI, USA). Barcode sequences of all *Helminthosporium* species currently available in GenBank, representative strains from other genera in Massarinaceae, and the outgroup taxon *Periconia pseudodigitata* (CBS 139699) were downloaded from the NCBI nucleotide database using the function read.GenBank integrated within the R package Analysis of Phylogenetics and Evolution (APE) [29].

The multiple sequence alignment was conducted using MAFFT version 7.310 [30] with options “--adjustdirectionaccurately--auto”, and the alignment results were further trimmed using trimAl version 1.4 [31] with the option “-gapthreshold 0.5”, which only allows 50% of taxa with a gap in each site. The best-fit nucleotide substitution models for each alignment dataset were selected using PartitionFinder version 2.1.1 [32] under the Corrected Akaike Information Criterion (AICC).

Maximum Likelihood (ML) and Bayesian analysis were conducted based on individual and combined datasets. Five alignment datasets of SSU, ITS, LSU, *TEF1*, and *RPB2* were concatenated using an in-house python script for multi-locus phylogenetic analysis. ML phylogenetic trees were obtained using the IQ-TREE version 2.0.3 [33], and the topology was evaluated using 1000 ultrafast bootstrap replicates. The Bayesian analysis was conducted using parallel MrBayes version 3.2.7a [34]. Two different runs with 20 million generations and four chains were executed, and the initial 25% of sample trees were treated as burn-in. Tracer version 1.7.1 [35] was used to confirm that the MCMC runs reached convergence with all ESS values above 200. Then, the ML tree was annotated by TreeAnnotator version 2.6.6 implemented in BEAST version 2.6.6 [36] based on MrBayes MCMC trees with no discard of burn-in, and no posterior probability limit. The ML trees were visualized using ggtree [37] and further edited in Adobe Illustrator version 16.0.0.

## 3. Results

### 3.1. Molecular Phylogeny

Five barcode sequences were obtained successfully except for *RPB2* of *H. velutinum* (UESTCC 22.0022) and *H. chengduense* sp. nov. Newly generated sequences were deposited in GenBank and the accession numbers are listed in Table 2. The combined dataset (ITS:1-557, SSU:558-1594, LSU:1595-2485, *TEF1*:2459-3719, *RPB2*: 3720-4836) was composed of 1744 distinct patterns, 1138 parsimony-informative sites, 325 singleton sites and 3373 constant sites. Five single-locus datasets ITS, SSU, LSU, *RPB2*, and *TEF1* contained 258, 69, 106, 353, and 357 parsimony informative sites, respectively. The best-fit evolution models were GTR+I+G for the ITS, LSU, *TEF1* and *RPB2* partitions and HKY+G for the SSU partition.

**Figure 1:**
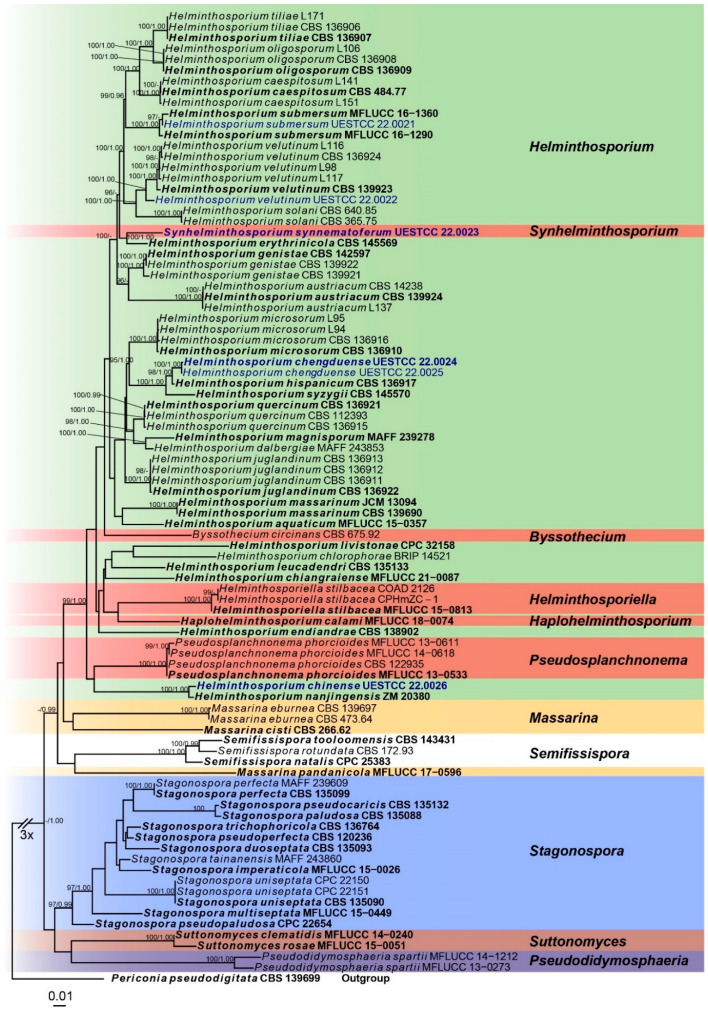
Phylogram of the best ML tree based on a combined dataset (SSU, ITS, LSU, *TEF1*, and *RPB2*) of Massarinaceae. Novel isolates are indicated in dark blue. Isolates from type materials are in bold. The ML ultrafast bootstrap values/Bayesian PP greater than 95%/0.95 are indicated at the respective nodes. The tree is rooted with *Periconia pseudodigitata* (CBS 139699) (Periconiaceae, Pleosporales).

The best-scoring ML consensus tree (lnL = −21,360.862) with ultrafast bootstrap values from ML analyses and posterior probabilities from MrBayes analysis at the node is shown in Figure 1. *Helminthosporium* species are mixed with species of other genera, viz. *Byssothecium*, *Pseudosplanchnonema*, and *Haplohelminthosporium*, suggesting the genus is polyphyletic. Six newly obtained *Helminthosporium* isolates represent five different clades. *Helminthosporium submersum* (UESTCC 22.0021) clustered with two *H. submersum* isolates (MFLUCC 16-1360 and MFLUCC 16-1290). *Helminthosporium velutinum* (UESTCC 22.0022) and the five other *H. velutinum* strains including the epi-type (CBS 139923) are grouped into a statistically well-supported clade (100/1.00). *Synhelminthosporium synnematoferum* (UESTCC 22.0023) is separate from *H. erythrinicola* (CBS 145569) with strong statistical support (100/1.00). Two isolates (UESTCC 22.0025 and UESTCC 22.0024) of *H. chengduense* form a distinct clade sister to *H. hispanicum* with high support values (98/1.00). *Helminthosporium chinense* (UESTCC 22.0026) is a sister to *H. nanjingensis* (ZM 20380) with strong statistical support (100/1.00).

### 3.2. Taxonomy

**Figure 2:**
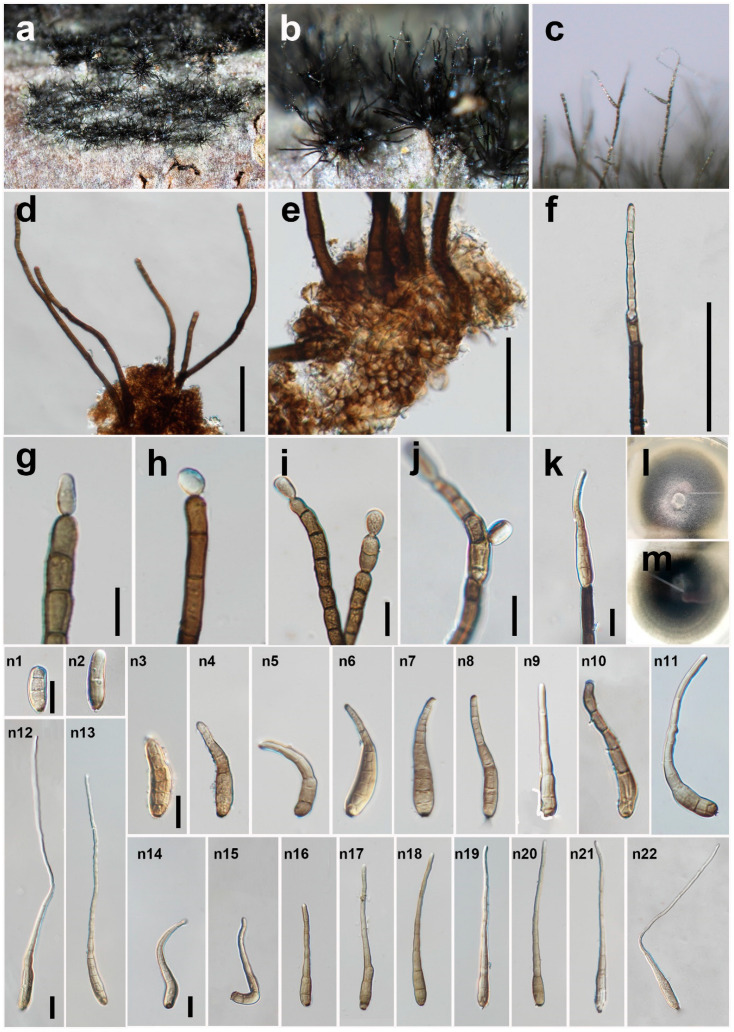
*Helminthosporium chengduense* (HKAS 124016, holotype). (**a**,**b**) Colonies on the natural substrate; (**c**) Conidiophores with apical and lateral conidia; (**d**) Conidiophores and stroma cells; (**e**) Conidiophore bases and stroma cells; (**f**) Conidiophore; (**g**–**i**) Conidiophore with young apical conidia; (**j**) Conidiophore with a young lateral conidium; (**k**) Conidiophore with an apical conidium; (**l**,**m**) Culture on PDA after 2 weeks (back and forth); (**n1**–**n22**) Conidia. Scale bars: (**d**,**f**) = 100 μm; (**e**,**g**,**i**–**k**,**n1**,**n3**,**n12**,**n14**) = 20 μm. Scale bar (**g**) applies to (**h**); (**n1**) applies to (**n2**); (**n3**) applies to (**n4**–**n11**); (**n12**) applies to (**n13**); (**n14**) applies to (**n15**–**n22**).

***Helminthosporium chengduense*** Y.P. Chen & Maharachch., sp. nov. (Figure 2).

*MycoBank*: MB 844416

*Etymology*: The name refers to Chengdu, the city where the fungus was collected.

*Saprobic* on decaying wood in a damp environment. **Sexual morph:** Unknown. **Asexual morph:** *Colony* on natural substrate punctiform, black, hairy. *Mycelium* mostly immersed, towards the surface forming stroma-like aggregations of light to dark brown pseudoparenchymatous cells. *Conidiophores* 133–391 μm long (x = 252, n = 40), 8–15 μm wide (x = 12, n = 40) at the base, tapering to 7–11 μm (x = 9, n = 40) at the apex, arising solitarily or in small groups from the stroma cells, erect, simple, straight or flexuous, thick-walled, subcylindrical, smooth, pale to dark brown, paler near the apex, with well-defined small pores at the apex and rarely laterally beneath the upper 1–2 septa. *Conidiogenous cells* mono- to poly-tretic, cylindrical, integrated, terminal and intercalary, pale brown to brown, secession schizo-lytic. *Conidia* 41–251 × 8–13 μm (x = 120 × 10, n = 60), tapering to 2–6 μm (x = 4, n = 55) at the distal end, with a blackish-brown 2–5 μm wide (x = 3, n = 25) scar at the base, obclavate, straight, flexuous, sigmoid, lunate or uncinate, thin-walled, smooth, grey-white to pale brown, 3–16-distoseptate (n = 52), with angular lumina; wall up to 2–4 μm thick (x = 3, n = 68). 

*Material examined*: China, Sichuan Province, Chengdu City, Yunqiao Wetland, on decaying branch of unidentified host, N 30°52′32, E 103°53′23, elevation 570 m, 10 July 2021, Y.P. Chen, YQ 071048H (HKAS 124016, holotype), culture ex-type UESTCC 22.0024 = YQ 071,048 = CGMCC 3.23575; *ibid.*, YQ 071047H (HUEST 22.0025, isotype), culture ex-isotype UESTCC 22.0025 = YQ 071047.

*Culture characteristics*: Colony on PDA 53 mm diam after 2 weeks in an incubator under dark conditions at 20 °C, pale green, irregular circular, surface velvety, with white and denser mycelium at the center, with creamy white, entire margin; reverse dark green at the center, pale green at the periphery, with growth rings.

*Notes*: The phylogenetic tree shows that the isolate UESTCC 22.0024 clusters with the ex-type strain of *H. hispanicum* (CBS 136917) [8]. However, the isolate UESTCC 22.0024 significantly differs from the holotype in the length of conidiophores (133–391 μm vs. 130–540 μm), the size of conidia (41–251 × 8–13 μm vs. 69–130 × 17–24 μm), the wall thickness of angular lumina (2–4 μm vs. 7 μm). In addition, *H. hispanicum* is fungicolous and grows on old conidiomata of *Juglanconis juglandina* [8], whereas *H. chengduense* (UESTCC 22.0024) is saprobic on decaying wood in damp environments. Konta et al. [7] summarized the morphological characteristics of 216 *Helminthosporium* species. Among them, only *H. asterinum*, *H. longisinuatum*, and *H. makilingense* produce larger conidia than *H. chengduense* UESTCC 22.0024. The conidia of *H. chengduense* (UESTCC 22.0024) are much shorter and narrower than *H. asterinum* (500–600 × 80 μm) [38]; shorter than *H. longisinuatum* (65–1000 μm) [39], but with longer conidiophores (133–391 μm vs. 20–75 μm) and a smaller number of distosepta (3–16 vs. 9–22); shorter than *H. makilingense* (100–300 μm) [40]. Considering the significant differences in morphology and molecular data, we introduce the isolate UESTCC 22.0024 as a new species *H. chengduense*.

**Figure 3:**
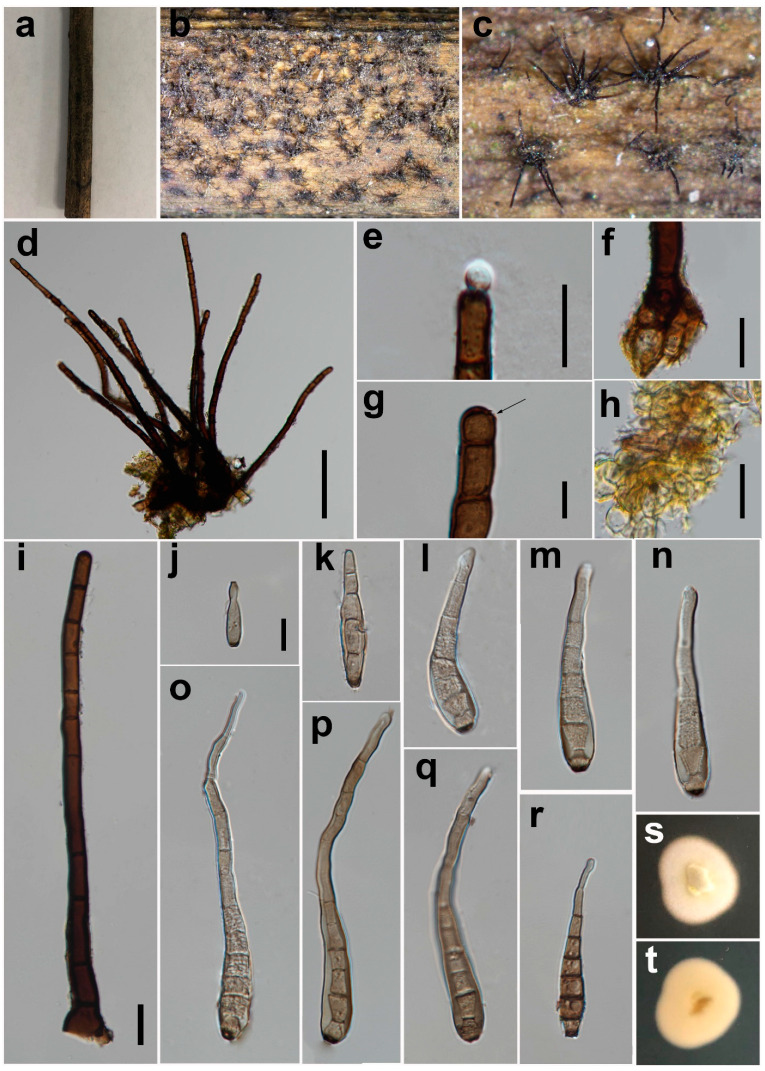
*Helminthosporium chinense* (HKAS 124017, holotype). (**a**) Natural substrate; (**b**,**c**) Colonies on the substrate; (**d**) Colony and conidiophores; (**e**) Conidiophore with an apical conidium; (**f**) Conidiophore base and stroma cells; (**g**) Conidiophore with an apical pore; (**h**) Stroma cells; (**i**) Conidiophore; (**j**) Young conidium; (**k**–**q**) Conidia; (**r**) Old conidium. (**s**,**t**) Front view and back view of culture on PDA after 2 weeks. Scale bars: (**d**) = 100 μm, (**e**,**g**–**i**) = 20 μm, (**f**,**j**) = 10 μm. Scale bar j applies to (**k**–**r**).

***Helminthosporium chinense*** Y.P. Chen & Maharachch., sp. nov. (Figure 3).

*MycoBank*: MB 844417

*Etymology*: The name refers to China, the country where the fungus was collected.

*Saprobic* on decaying wood in damp environment. **Sexual morph:** Unknown. **Asexual morph:** *Colony* on natural substrate effuse, black, hairy. *Mycelium* mostly immersed, towards the surface forming stroma-like aggregations of light to brown pseudoparenchymatous cells. *Conidiophores* 214–461 μm long (x = 326, n = 40), 8–16 μm wide (x = 11, n = 38) at the base, tapering to 6–10 μm (x = 8, n = 38) at the apex, arising solitarily or in fascicles from the stroma cells, erect, simple, straight or flexuous, thick-walled, subcylindrical, smooth, pale to dark brown, with well-defined small pores at the apex and rarely laterally beneath the upper 1–5 septa. *Conidiogenous cells* mono- to poly-tretic, cylindrical, integrated, terminal and intercalary, brown, secession schizo-lytic. *Conidia* 42–109 × 5–11 μm (x = 61 × 8, n = 35), tapering to 2–6 μm (x = 4, n = 35) at the distal end, with a blackish-brown 3–5 μm wide (x = 4, n = 27) scar at the base, obclavate, straight or flexuous, thin-walled, smooth, pale gray to brown, 4–10-distoseptate, with angular lumina; wall up to 1–3 μm thick (x = 2, n = 36).

*Material examined*: China, Sichuan Province, Chengdu City, Yunqiao Wetland, on decaying branch of palm trees, N 30°52′32, E 103°53′23, elevation 570 m, 10 July 2021, Y.P. Chen, YQ 071005H (HKAS 124017, holotype), culture ex-type UESTCC 22.0026 = YQ 071,005 = CGMCC 3.23570.

*Culture characteristics*: Colony on PDA 31 mm diam after 2 weeks in an incubator under dark condition at 20 °C, white, irregular circular, surface velvety, with a clear margin; reverse white, with clear margin.

*Notes*: The phylogenetic tree shows that the isolate UESTCC 22.0026 clusters with the ex-type strain (ZM 20380) of *H. nanjingensis*, which was introduced by Wang et al. [41] from dead branches of an unidentified tree in Nanjing City, Jiangsu Province, China. Our collection (HKAS 124017) shares similar morphological characteristics in the shape and color of conidiophores and conidia with the holotype (HSAUP_02_0198) [41] of *H. nanjingensis* on natural substrate. However, it differs from *H. nanjingensis* by having significantly shorter conidia (42–109 μm vs. 64.5–170.5 μm) and smaller number of disto-septa (4–10 vs. 6–17) [41]. The BLASTn analysis of ITS of our isolate UESTCC 22.0026 showed 98% identity (446/453 bp, no gap) with ex-type strain (ZM 20380) of *H. nanjingensis*. *Helminthosporium nanjingensis* produced yellow-green pigment on PDA media [41], but the isolate UESTCC 22.0026 does not produce pigment on PDA. Only the ITS sequence is available for *H. nanjingensis*. Therefore, we cannot compare the sequence difference of other barcodes. Thus, considering the difference in morphology and the ability to produce pigments, we describe the isolate UESTCC 22.0026 as a new species *H. chinense*.

**Figure 4:**
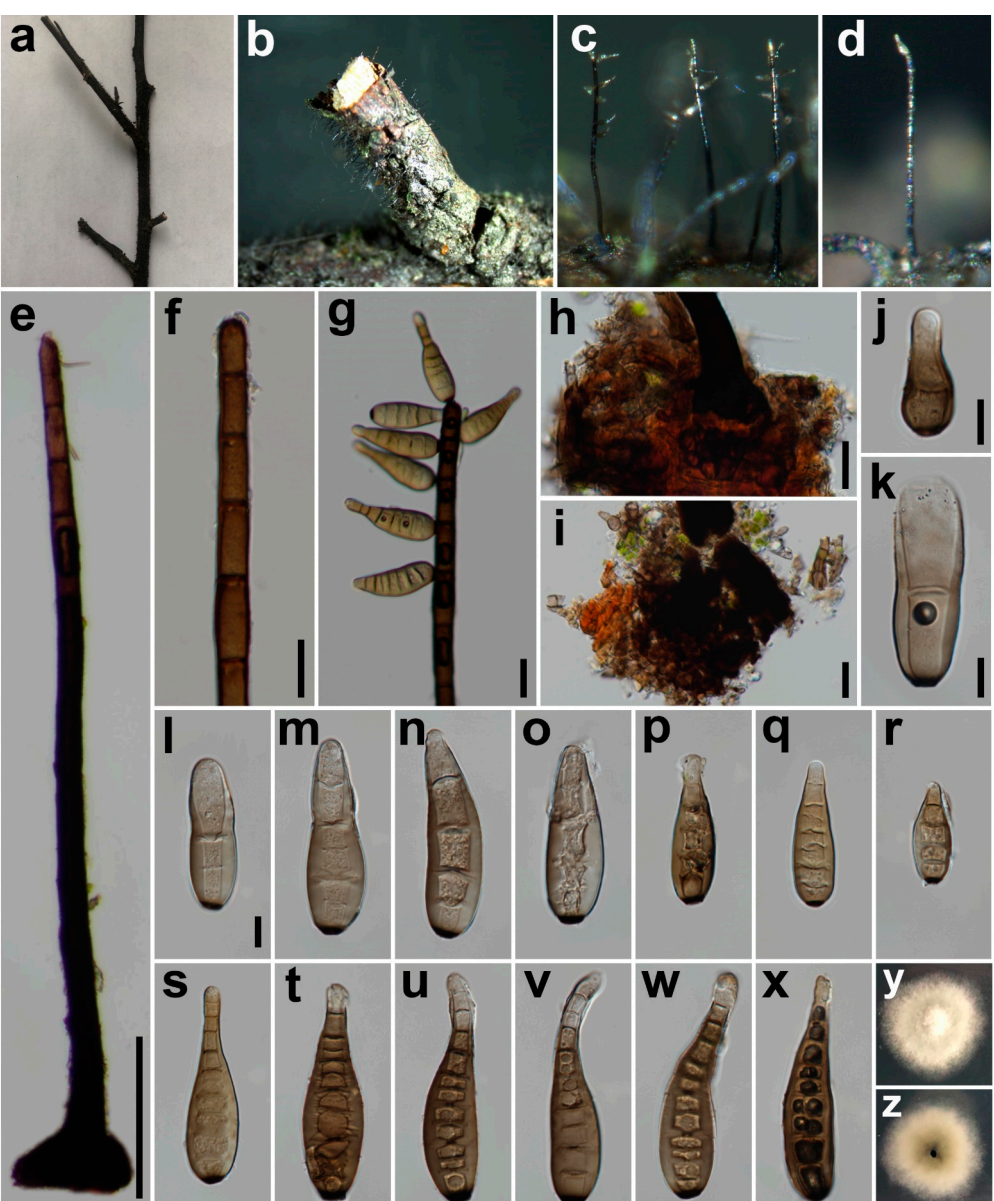
*Helminthosporium submersum* (HUEST 22.0021). (**a**) Natural substrate; (**b**–**d**) Colonies on natural substrates; (**e**) Conidiophore; (**f**) Conidiophore apex; (**g**) Conidiophore, conidiophore apex, apical conidia and lateral conidia; (**h**,**i**) Conidiophore base and stroma cells; (**j**–**x**) Conidia; (**y**,**z**) Front view and back view of a colony on PDA after 2 weeks. Scale bars: (**e**–**g**) = 100 μm, (**h**,**i**) = 20 μm, (**j**–**l**) = 10 μm. Scale bar (**l**) applies to (**m**–**x**).

***Helminthosporium submersum*** Z.L. Luo & H.Y. Su, Phytotaxa 348(4): 274 (2018) (Figure 4).

*Saprobic* on decaying wood in damp environment. **Sexual morph:** unknown. **Asexual morph:** *Colony* on natural substrate superficial, effuse, hairy, dark brown to black, glistening. *Mycelium* mostly immersed, composed of septate, unbranched, smooth, thick-walled hyphae, on the bark stroma-like aggregations of light to black dark brown pseudoparenchymatous cells at the hyphae base. *Conidiophores* mono-nematous, erect, simple, straight or flexuous, unbranched, smooth, thick-walled, subcylindrical, pale to dark brown, paler near the apex, 130–570 μm long, 15–31 μm wide (x = 21, n = 20) at the base, tapering to 9–12 μm (x = 10, n = 20) at the apex, with well-defined small pores at the apex and laterally beneath the upper 1–5 septa. *Conidiogenous cells* mono- to poly-tretic, integrated, terminal and intercalary, cylindrical, pale brown, secession schizo-lytic. *Conidia* acropleurogenous, simple, obclavate, straight or flexuous, thin-walled, smooth, grey-brown to brown, 49–86 × 14–25 μm (x = 70 × 18, n = 35), tapering to 6–12 μm (x = 8, n = 35) at the distal end, with a blackish-brown 3–6 μm wide (x = 5, n = 40) scar at the base, 1–12-distoseptate (n = 30), with angular lumina; wall up to 2–4 μm thick (x = 3, n = 35).

*Material examined*: China, Sichuan Province, Dujiangyan City, Longchi National Forest Park, on decaying branch of an unidentified host, N 31°6′37, E 103°33′55, elevation 1168 m, 19 September 2021, W. Tian, Sarah 08_3H (HUEST 22.0021), living culture UESTCC 22.0021 = Sarah 08_3 = CGMCC 3.23571.

*Culture characteristics*: Colony on PDA 19 mm diam after 2 weeks in an incubator under dark conditions at 20 °C, white, irregular circular, surface velvety, with denser mycelium at the center and becoming sparser towards the edge, with unclear margin; reverse pale green at the center, with unclear white margin.

*Notes*: The phylogenetic tree showed that our isolate (UESTCC 22.0021) from decaying wood in a damp environment clustered with the ex-type strain (MFLUCC 16–1360) of *H. submersum*, which was introduced by Zhao et al. [42] from submerged wood in freshwater. Morphologically, our collection fits well with *H. submersum* by having effuse, velvety, dark brown or black colonies, mono-nematous, straight or flexuous, unbranched, pale to dark brown conidiophores, and similar size (49–86 × 14–25 μm vs. 41–55 × 14.5–18.5 μm) conidia [42]. Based on the overlapping morphological characteristics and the multi-locus phylogenetic tree, we identify our isolate as *H. submersum*. This is the first report of *H. submersum* isolated from decaying wood in terrestrial habitats.

**Figure 5:**
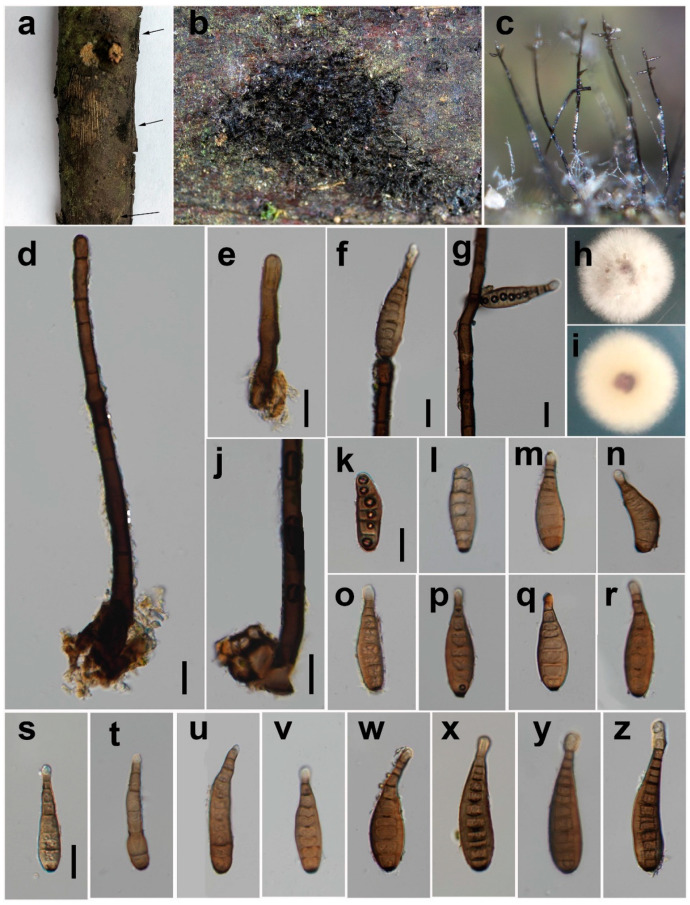
*Helminthosporium velutinum* (HUEST 22.0022). (**a**,**b**) Colonies on the natural substrate; (**c**) Conidiophores with apical and lateral conidia; (**d**,**e**,**j**) Conidiophores with stroma cells; (**f**) Conidiophore apex with an apical conidium; (**g**) Conidiophore with a lateral conidium; (**h**,**i**) Front view and back view of culture on PDA after 1 week; (**k**–**z**) Conidia. Scale bars: (**d**–**g**,**j**,**k**,**s**) = 20 μm. Scale bar k applies to (**l**–**r**); (**s**) applies to (**t**–**z**).

***Helminthosporium velutinum*** Link [as ‘Helmisporium’], Mag. Gesell. naturf. Freunde, Berlin 3(1–2): 10, Table 1:9 (1809) (Figure 5).

*Saprobic* on decaying wood in damp environment. **Sexual morph:** Unknown. **Asexual morph:** *Colony* on natural substrate effuse, black, hairy, glistening. *Mycelium mostly immersed*, on the surface forming stroma-like aggregations of light to dark brown pseudoparenchymatous cells. *Conidiophores* 343–941 μm long (x = 715, n = 20), 12–30 μm wide (x = 20, n = 20) at the base, tapering to 9–16 μm (x = 107, n = 20) at the apex, arising solitarily or in fascicles from the stroma cells, erect, simple, straight or flexuous, cylindrical, thick-walled, smooth, dark to blackish brown, paler near the apex, with well-defined small pores at the apex and rarely laterally beneath the upper 1–11 septa. *Conidiogenous cells* mono- to poly-tretic, cylindrical, integrated, terminal and intercalary, brown, secession schizo-lytic. Conidia 53–99 × 13–22 μm (x = 76 × 17, n = 36), tapering to 5–8 μm (x = 7, n = 36) at the distal end, with a blackish-brown 3–5 μm wide (x = 4, n = 33) scar at the base, obclavate, straight or flexuous, thin-walled, smooth, pale brown, 6–13-distoseptate, with angular lumina; wall up to 2–5 μm thick (x = 4, n = 43).

*Material examined*: China, Sichuan Province, Chongzhou City, Baiyungou, on decaying branch of an unidentified host, N 30°47′56, E 103°24′15, elevation 990 m, 27 September 2021, Y.P. Chen, BY 14_2H (HUEST 22.0022), living culture UESTCC 22.0022 = BY 14_2 = CGMCC 3.23572.

*Culture characteristics*: Colony on PDA 19 mm after 2 weeks in an incubator under dark conditions at 20 °C, creamy white, irregular circular, surface velvety, with denser mycelium at the center and becoming sparser towards the edge, with clear margin; reverse white, pale green in the center, with clear margin.

*Notes*: The phylogenetic tree showed that the isolate HUEST 22.0022 clustered with the isolates of *H. velutinum*. *Helminthosporium velutinum*, the type of the genus, is a well-known and most commonly recorded species [8]. It has been recorded mainly from woody substrates, and it is known for more than 100 host records [43]. Zhu et al. [12] first reported *H. velutinum* from a freshwater habitat in China, which is a less common habitat for this species. Our collection (HUEST 22.0022) displays similar morphological characteristics with the type of *H. velutinum* in the shape and color of colonies, conidiophores, conidiogenous cells, and conidia on the natural substrate [8]. We identified the isolate UESTCC 22.0022 as *H. velutinum*, a new record from terrestrial habitats in china considering similar morphological characteristics.

***Synhelminthosporium*** Y.P. Chen & Maharachch., gen. nov.

*MycoBank*: MB 844418

*Etymology*: Syn = together with its close phylogenetic relationship with *Helminthosporium*.

*Saprobic* on decaying wood in damp environment. **Sexual morph:** Unknown. **Asexual morph:** *Colony* on natural substrate evenly distributed, hairy, glistening. *Mycelium mostly immersed*. *Conidiophores* synnematous, compact, erect, straight or flexuous, unbranched, septate, smooth, subcylindrical, brown to dark brown, separated from the main body near the apex. *Conidiogenous cells* mono-tretic, cylindrical, integrated, terminal, brown, secession schizo-lytic. *Conidia* obclavate, straight or flexuous, smooth, pale brown, distoseptate, with angular lumina.

Type species: Synhelminthosporium synnematoferum Y.P. Chen & Maharachch. sp. nov.

*Notes*: The genus *Synhelminthosporium* is introduced based on the new species *S. synnematoferum*. Both BLASTn analysis results of five barcode sequences (SSU, ITS, LSU, *TEF1*, and *RPB2*) and multi-locus phylogenetic analyses showed that *Synhelminthosporium* is distinct and phylogenetically close to *Helminthosporium*. *Synhelminthosporium* differs from *Helminthosporium* by having synnematous conidiophores, which is a character only presented in *Helminthosporiella* within Massarinaceae [7,44]. Phylogenetic analysis shows that *Synhelminthosporium* is different from *Helminthosporiella*. In addition, *Helminthosporiella* shows catenate conidia, but this character is absent in *Synhelminthosporium*. Based on distinguishing morphological characteristics and multi-locus phylogenetic analyses, we introduce a new genus *Synhelminthosporium* to accommodate the new species *S. synnematoferum* in Massarinaceae.

**Figure 6:**
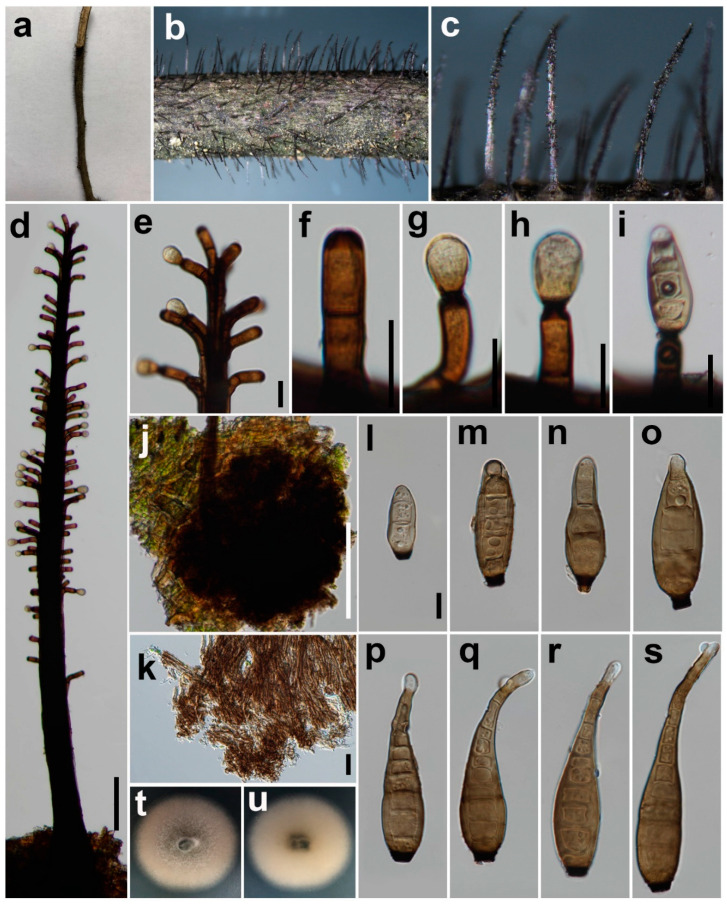
*Synhelminthosporium synnematoferum* (HKAS 124015, holotype). (**a**–**c**) Colonies on natural substrates; (**d**) Synnematous conidiophores; (**e**) Conidiophores with apical conidia; (**f**) Conidiogenous cell with an apical pore; (**g**–**i**) Conidiogenous cells with apical conidia; (**j**,**k**) Conidiophore bases; (**l**) Young conidium; (**m**–**s**) Conidia; (**t**,**u**) Front view and back view of culture on PDA after 1 week. Scale bars: (**d**,**j**) = 100 μm, (**e**–**i**,**k**) = 20 μm, (**l**) = 10 μm. Scale bar (**l**) applies to (**m**–**s**).

***Synhelminthosporium synnematoferum*** Y.P. Chen & Maharachch., sp. nov. (Figure 6).

*MycoBank*: MB 844419

*Etymology*: The name refers to the synnematous conidiophores.

*Saprobic* on decaying wood in damp environment. **Sexual morph:** Unknown. **Asexual morph:** *Colony* on natural substrate evenly distributed, hairy, glistening. *Conidiophores* 700–1500 μm long, synnematous, compact, erect, straight or flexuous, unbranched, septate, smooth, subcylindrical, brown to dark brown, separated from the main body from the middle part up to near the apex. *Conidiogenous cells* mono-tretic, cylindrical, integrated, terminal, brown, secession schizo-lytic. *Conidia* 52–116 × 14–20 μm (x = 80 × 16, n = 36), tapering to 5–10 μm (x = 7, n = 36) at the distal end, with a blackish-brown 3–7 μm wide (x = 5, n = 36) scar at the base, obclavate, straight or flexuous, smooth, pale brown, 4–11-distoseptate (n = 35), with angular lumina; wall up to 2–4 μm thick (x = 3, n = 36).

*Material examined*: China, Sichuan Province, Pengzhou City, Huilonggou, on decaying branch of an unidentified host, N 31°11′6, E 103°54′56, elevation 1400 m, 28 July 2021, Y.P. Chen, HLG 072894H (HKAS 124015, holotype), culture ex-type UESTCC 22.0023 = HLG 072,894 = CGMCC 3.23574.

*Culture characteristics*: Colony 52 mm diam after 1 week in an incubator under dark condition at 20 °C, creamy white, irregular circular, surface velvety, with denser mycelium at the center and becoming sparser at the edge, with a clear margin; reverse creamy white, pale green at the center, with clear margin.

*Notes*: The best BLASTn matches of five barcode sequences (SSU, ITS, LSU, *TEF1*, and *RPB2*) of the isolate UESTCC 22.0023 belong to the genus *Helminthosporium*. The phylogenetic tree shows that our isolate (UESTCC 22.0023) clusters with the ex-type strain *H. erythrinicola* (CBS 145569). The BLASTn analysis of *H. erythrinicola* (CBS 145569) and *S. synnematoferum* (UESTCC 22.0023) shows 94% identity (538/570, 5 gaps) using ITS, 99% identity (820/831, 3 gaps) using LSU and 93% identity (822/884, no gap) using *RPB2*. *Helminthosporium erythrinicola* was introduced by Crous et al. [45], it is a typical *Helminthosporium* species with simple, multiseptated, unbranched, brown conidiophores, tretic conidiogenous cells, and acro-pleurogenous, clavate or obclavate, distoseptate conidia. Our collection (HKAS 124015) differs from the *H. erythrinicola* and other *Helminthosporium* species in having synnematous conidiophores. Synnematous conidiophores are only present in *Helminthosporiella* in Massarinaceae [7,44]. The isolate UESTCC 22.0023 is different from *Helminthosporiella* phylogenetically and morphologically. The absence of catenate conidia is the most prominent characteristic distinguishing *Synhelminthosporium synnematoferum* from *Helminthosporiella* species. The multi-locus phylogenetic tree shows the placement of *S. synnematoferum* (UESTCC 22.0023) within Massarinaceae, which is distinct from other known species.

## 4. Discussion

To date, there are 775 epithets of *Helminthosporium* (http://www.indexfungorum.org; accessed on 10 April 2022), whereas many of them are not congeneric with the generic type, and were reclassified into other groups in subsequent studies [46,47,48]. For instance, *H. cynodontis* was reassigned to the genus *Bipolaris* (Pleosporaceae, Pleosporales) due to the production of sympodial conidiogenous cells and by having darkly pigmented conidiogenous loci [46]. Recently, Konta et al. [7] accepted 216 *Helminthosporium* species, however many species are identified only based on morphological studies, and only 25 species have sequence data. The lack of a large amount of molecular data is mainly because most species were introduced before the advent of Sanger sequencing. Considering that numerous *Helminthosporium* species were characterized only based on morphological studies, it is likely that some of them belong to the same species or even to different genera. During phylogenetic analysis, abnormal long branches were observed in four *Helminthosporium* strains with incorrect taxonomic positions, viz. *H. asterinum* (CBS 203.35), *H. decacuminatum* (CBS 185.47), *H. anomalum* (CBS 161.27), and *H. gibberosporum* (CBS 200.32). These species were introduced and characterized before the 1950s [49,50,51,52], whereas the sequence data related to them were submitted to GenBank by Vu et al. [53]. BLASTn analyses of these sequences showed that the top hits of ITS and LSU sequences for *H. anomalum* (CBS 161.27) belong to *Bipolaris*, ITS and LSU sequences for *H. asterinum* (CBS 203.35) belong to *Kirschsteiniothelia*, ITS and LSU sequences for *H. decacuminatum* (CBS 185.47) and *H. gibberosporum* (CBS 200.32) belong to *Curvularia*. The present study introduces two new *Helminthosporium* species and a new genus *Synhelminthosporium* based on multi-locus phylogenetic analysis and morphological studies. This phylogeny needs to be expanded by re-examining type materials of old described *Helminthosporium*-like species without molecular data, collecting new fresh specimens, sequencing, and using multi-locus analysis to establish epi-types or neotypes as necessary. Our new species can be distinguished from all other *Helminthosporium* species by morphological features and multi-locus phylogenetic analysis, and thus we are confident that the newly introduced species are distinct.

Recent studies have no universally accepted standard in selecting barcodes for phylogenetic analysis. Boonmee et al. [54] introduced *H. chiangraiense* using ITS and LSU. Crous et al. [45] introduced *H. erythrinicola* and *H. syzygii* using ITS, LSU, and *RPB2*. Zhu et al. [12] introduced *H. aquaticum* using SSU, ITS, and LSU barcodes. Voglmayr and Jaklitsch [8] pointed out that only ITS and/or LSU sequences can be problematic in resolving the phylogeny of Massarinaceae. Other barcodes *RPB2* and *TEF1* were proposed in multi-gene phylogenetic analyses of Massarineae, as these barcodes usually significantly increase the phylogenetic resolution [8,11]. However, the majority of *Helminthosporium* species do not have *RPB2* and *TEF1* barcodes (19 species have SSU sequence data; 25 species have SSU sequence data; 23 species have LSU sequence data; 17 species have *RPB2* sequence data and 15 species have *TEF1* sequence data). The present study conducted both individual and combined phylogenetic analyses. ITS, *RPB2*, and *TEF1* barcodes offered more parsimony informative sites than SSU and LSU. In addition, more powerful resolution in delineating species and higher bootstrap support values for most clades were observed in single-gene ML trees (Figures S1 and S2), indicating that these barcodes are better in resolving genera in Massarineae than the other two barcodes.

## Figures and Tables

**Table 1:** Loci used in this study with the corresponding PCR primers and conditions.

Locus	PCR Primers	PCR: Thermal Cycles	Reference
ITS	ITS9mun/ITS4_KYO1	(94 °C: 30 s, 56 °C: 30 s, 72 °C: 30 s) × 35 cycles	[19]
LSU	LR0R/LR5	(94 °C: 30 s, 56 °C: 30 s, 72 °C: 1 min) × 35 cycles	[20,21]
SSU	PNS1/NS41	(94 °C: 30 s, 56 °C: 30 s, 72 °C: 1 min) × 35 cycles	[22]
*TEF1*	EF1-728F or EF1-983/EF1-2218R or TEF1LLErev	(94 °C: 30 s, 52 °C: 30 s, 72 °C: 1 min) × 35 cycles	[23,24,25]
*RPB2*	dRPB2-5f or RPB2-5F2/dRPB2-7r or fRPB2-7cR	(94 °C: 30 s, 52 °C: 30 s, 72 °C: 1 min) × 35 cycles	[26,27,28]

**Table 2:** Isolates and GenBank accessions used in the phylogenetic analyses.

Organism	Culture/Specimen No. ^1^	SSU ^2,3^	LSU	ITS	*RPB2*	*TEF1*
*Byssothecium circinans*	CBS 675.92	GU205235	GU205217	OM337536	DQ767646	GU349061
*Haplohelminthosporium calami*	MFLUCC 18-0074 ^HT^	MT928160	MT928156	MT928158	– ^2^	–
*Helminthosporiella stilbacea*	COAD 2126	–	–	MG668862	–	MG682500
*H. stilbacea*	MFLUCC 15-0813 ^HT^	MT928161	MT928157	MT928159	–	MT928151
*H. stilbacea*	CPHmZC-01	–	KX228355	KX228298	–	–
*Helminthosporium aquaticum*	MFLUCC 15-0357 = S-096 ^HT^	KU697310	KU697306	KU697302	–	–
*H. austriacum*	CBS 139924 = L132 ^HT^	KY984420	KY984301	KY984301	KY984365	KY984437
*H. austriacum*	CBS 14238 = L169	–	KY984303	KY984303	KY984367	KY984439
*H. austriacum*	L137	–	KY984302	KY984302	KY984366	KY984438
*H. caespitosum*	CBS 484.77 = L99 ^ET^	KY984421	JQ044448	JQ044429	KY984370	KY984440
*H. caespitosum*	L141	–	KY984305	KY984305	KY984368	–
*H. caespitosum*	L151	–	KY984306	KY984306	KY984369	–
***H. chengduense***	**UESTC 22.0024 = YQ 071048 = CGMCC 3.23575 ^HT^**	**ON557757**	**ON557745**	**ON557751**	**ON563073**	**ON600598**
***H. chengduense***	**UESTC 22.0025 = YQ 071047**	**ON557756**	**ON557744**	**ON557750**	**ON563072**	**ON600597**
*H. chiangraiense*	MFLUCC 21-0087 ^HT^	–	MZ538538	MZ538504	–	–
*H. chlorophorae*	BRIP 14521	–	–	AF120259	–	–
*H. dalbergiae*	MAFF 243853 = H 4628 = TS 36	AB797231	AB807521	LC014555	–	AB808497
*H. endiandrae*	CBS 138902 = CPC 22194 ^HT^	–	KP004478	KP004450	–	–
*H. erythrinicola*	CPC 35291 = CBS 145569 ^HT^	–	MK876432	NR_165563	MK876486	–
*H. genistae*	CBS 142597 = L142 ^ET^	–	KY984310	KY984310	KY984374	–
*H. genistae*	CBS 139922 = L129	KY984423	KY984309	KY984309	KY984373	–
*H. genistae*	CBS 139921 = L128	KY984422	KY984308	KY984308	KY984372	–
*H. hispanicum*	CBS 136917 = L109 ^HT^	KY984424	KY984318	KY984318	KY984381	KY984441
*H. juglandinum*	CBS 136922 = L118 ^HT^	–	KY984321	KY984321	KY984384	KY984444
*H. juglandinum*	CBS 136911 = L97	KY984425	KY984322	KY984322	KY984385	KY984445
*H. juglandinum*	CBS 136912 = L101	–	KY984319	KY984319	KY984382	KY984442
*H. juglandinum*	CBS 136913 = L102	–	KY984320	KY984320	KY984383	KY984443
*H. leucadendri*	CBS 135133 = CPC 19345 ^HT^	–	KF251654	KF251150	KF252159	KF253110
*H. livistonae*	CPC 32158 = CBS 144413 ^HT^	–	NG_064539	NR_160348	–	–
*H. magnisporum*	MAFF 239278 = H 4627 = TS 33 ^HT^	AB797232	AB807522	AB811452	–	AB808498
*H. massarinum*	CBS 139690 = JCM 13095 = MAFF 239605 = KT 1564 ^HT^	AB797234	AB807524	AB809629	–	AB808500
*H. massarinum*	JCM 13094 = MAFF 239604 = KT 838 ^EP^	AB797233	AB807523	AB809628	–	AB808499
*H. microsorum*	CBS 136910 = L96 ^ET^	KY984427	KY984329	KY984329	KY984390	KY984448
*H. microsorum*	L94	KY984426	KY984327	KY984327	KY984388	KY984446
*H. microsorum*	CBS 136916 = L108	–	KY984323	KY984323	KY984386	–
*H. microsorum*	L95	–	KY984328	KY984328	KY984389	KY984447
*H. nanjingensis*	HHAUF020380 = ZM020380	–	–	KF192322	–	–
*H. oligosporum*	CBS 136909 = L93 ^ET^	–	KY984333	KY984333	KY984394	KY984451
*H. oligosporum*	CBS 136908 = L92	KY984428	KY984332	KY984332	KY984393	KY984450
*H. oligosporum*	L106	–	KY984330	KY984330	KY984391	KY984449
*H. quercinum*	CBS 136921 = L90 ^HT^	KY984429	KY984339	KY984339	KY984400	KY984453
*H. quercinum*	CBS 112393	–	KY984334	KY984334	KY984395	KY984452
*H. quercinum*	CBS 136915 = L107	–	KY984336	KY984336	KY984397	–
*H. solani*	CBS 365.75	KY984430	KY984341	KY984341	KY984402	KY984455
*H. solani*	CBS 640.85	–	KY984342	KY984342	KY984403	–
*H. submersum*	MFLUCC 16-1360 ^HT^	MG098796	MG098787	–	–	MG098586
*H. submersum*	MFLUCC 16-1290 ^PT^	MG098797	MG098788	MG098780	MG098592	MG098587
***H. submersum***	**UESTCC 22.0021 = Sara 08_3 = CGMCC 3.23571**	**ON557759**	**ON557747**	**ON557753**	**ON563075**	**ON600600**
*H. syzygii*	CPC 35312 = CBS 145570 ^HT^	–	MK876433	NR_165564	MK876487	–
*H. tiliae*	CBS 136907 = L88 ^ET^	KY984431	KY984345	KY984345	KY984406	KY984457
*H. tiliae*	CBS 136906 = L87	–	KY984344	KY984344	KY984405	–
*H. tiliae*	L171	–	KY984343	KY984343	KY984404	KY984456
*H. velutinum*	CBS 139923 = L131 ^ET^	KY984432	KY984352	KY984352	KY984413	KY984463
*H. velutinum*	L98	KY984433	KY984359	KY984359	KY984417	KY984466
*H. velutinum*	CBS 136924 = L115	–	KY984347	KY984347	KY984408	KY984458
*H. velutinum*	L116	–	KY984348	KY984348	KY984409	KY984459
*H. velutinum*	L117	–	KY984349	KY984349	KY984410	KY984460
***H. velutinum***	**UESTCC 22.0022 = BY 14_2 = CGMCC 3.23572**	**ON557761**	**ON557749**	**ON557755**	–	**ON600602**
***H. chinense***	**UESTCC 22.0026 = YQ 071,005 = CGMCC 3.23570 ^HT^**	**ON557760**	**ON557748**	**ON557754**	–	**ON600601**
*Massarina cisti*	CBS 266.62 = JCM 14140 ^HT^	AB797249	AB807539	LC014568	FJ795464	AB808514
*M. eburnea*	CBS 473.64	GU296170	GU301840	OM337528	GU371732	GU349040
*M. eburnea*	CBS 139697 = JCM 14422 = H 3953	AB521718	AB521735	LC014569	–	AB808517
*M. pandanicola*	MFLUCC 17-0596 = KUMCC 17-0293 ^HT^	MG646979	MG646947	MG646958	–	MG646986
*Periconia pseudodigitata*	KT 1395 = HHUF 29370 = CBS 139699 = JCM 13166 = MAFF 239676 ^HT^	NG_064850	NG_059396	NR_153490	–	AB808540
*Pseudodidymosphaeria spartii*	MFLUCC 13-0273	KP325438	KP325436	KP325434	–	–
*P. spartii*	MFLUCC 14-1212	KP325439	KP325437	KP325435	–	–
*Pseudosplanchnonema phorcioides*	L16 = CBS 122935	KY984434	KY984360	KY984360	KY984418	KY984467
*P. phorcioides*	MFLUCC 13-0533 = CGMCC 3.17583	KM875455	KM875454	–	–	–
*P. phorcioides*	MFLUCC 13-0611	KP683377	KP683376	KP683375	–	–
*P. phorcioides*	MFLUCC 14-0618	KP683374	KP683373	KP683372	–	–
*Semifissispora natalis*	CPC 25383 = CBS 140659 ^HT^	–	KT950858	KT950846	–	KT950878
*S. rotundata*	CBS 172.93 = CPC 549	–	KT950859	KT950847	–	–
*S. tooloomensis*	CBS 143431 = CPC 31680 ^HT^	–	NG_058526	NR_156674	–	–
*Stagonospora duoseptata*	CBS 135093 = S618 ^HT^	–	KF251758	KF251255	KF252260	KF253205
*S. imperaticola*	MFLUCC 15-0026 = ICMP 21563 ^HT^	KY706138	KY706133	KY706143	KY706149	KY706146
*S. multiseptata*	MFLUCC 15-0449 = ICMP 21562 ^HT^	–	NG_068239	NR_165854	–	–
*S. paludosa*	CBS 135088 = S601 ^NT^	–	KF251760	KF251257	KF252262	KF253207
*S. perfecta*	KT 1726A = JCM 13099 = MAFF 239609	AB797289	AB807579	AB809642	–	AB808555
*S. perfecta*	CBS 135099 = S656 ^HT^	–	KF251761	KF251258	KF252263	–
*S. pseudocaricis*	CBS 135132 = S610 ^HT^	–	KF251763	KF251259	KF252265	KF253210
*S. pseudopaludosa*	CPC 22654 = CBS 136424 ^HT^	–	NG_058052	NR_137840	–	–
*S. pseudoperfecta*	CBS 120236 = JCM 13097 = MAFF 239607 ^HT^	AB797287	AB807577	AB809641	–	AB808553
*S. tainanensis*	KT 1866 = MAFF 243860	AB797290	AB807580	AB809643	–	AB808556
*S. trichophoricola*	CBS 136764 = D652 ^HT^	–	NG_058081	NR_156586	KJ869232	–
*S. uniseptata*	CBS 135090 = S611 ^HT^	–	KF251767	KF251264	KF252269	–
*S. uniseptata*	S607 = CPC 22151	–	KF251768	KF251265	KF252270	KF253213
*S. uniseptata*	S608 = CPC 22150	–	KF251769	KF251266	KF252271	KF253214
*Suttonomyces clematidis*	MFLUCC 14-0240 = GUCC 18	KP842920	KP842917	–	–	–
*S. rosae*	MFLUCC 15-0051 ^HT^	MG829185	MG829085	MG828973	–	–
***Synhelminthosporium synnematoferum***	**UESTCC 22.0023 = HLG 072894 = CGMCC 3.23574 ^HT^**	**ON557758**	**ON557746**	**ON557752**	**ON563074**	**ON600599**

^1^ Isolates from type materials are marked with ET (epi-type), HT (holotype), NT (neotype), and PT (paratype). ^2^ Missing sequences are indicated by “–”. ^3^ Newly generated sequences are in bold.

## Data Availability

All sequence data are available in NCBI GenBank following the accession numbers in the manuscript.

## Supplementary Materials

The following supporting information can be downloaded at: https://www.mdpi.com/article/10.3390/jof8070712/s1, Figure S1: Single-gene trees. Novel isolates are indicated in dark blue. Type isolates are in bold. The ML ultrafast bootstrap values/Bayesian PP greater than 95%/0.95 are indicated at the respective nodes. The tree is rooted with *Periconia pseudodigitata* (CBS 139699) (Periconiaceae, Pleosporales). Figure S2: Comparisons of the single-gene ML trees and the multi-locus ML tree in delineating species. (a) The proportion of highly supported internal nodes, bootstrap values ≥ 95%. Protein-coding barcodes *TEF1* and *RPB2* offer higher proportions of highly supported internal nodes than three nuclear ribosomal regions (b) Pairwise tree distances. The smaller the value, the higher the similarity in topology.
