# Supplementary figures and images for: Synhelminthosporium gen. et sp. nov. and Two New Species of Helminthosporium (Massarinaceae, Pleosporales) from Sichuan Province, China
Source: J Fungi (Basel). 2022 Jul 5;8(7):712. doi: 10.3390/jof8070712 (PMC9316862; doi:10.3390/jof8070712)

a)

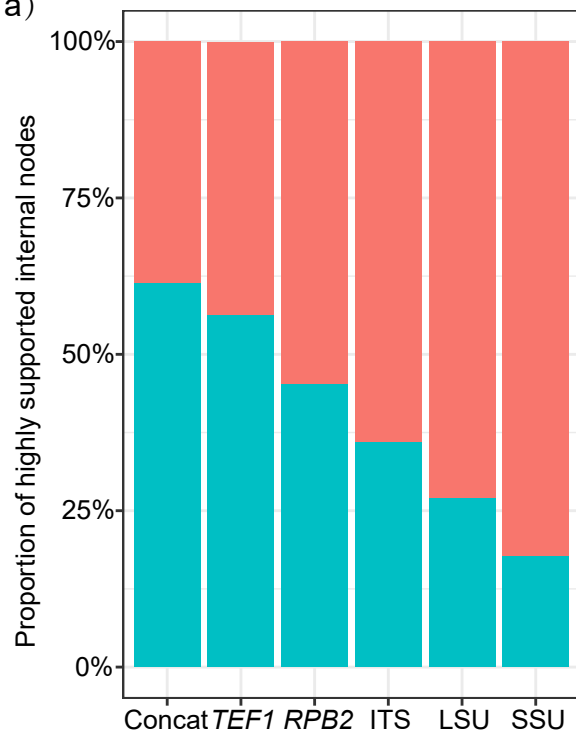

Support values

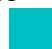

Above95

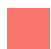

Below95

b)

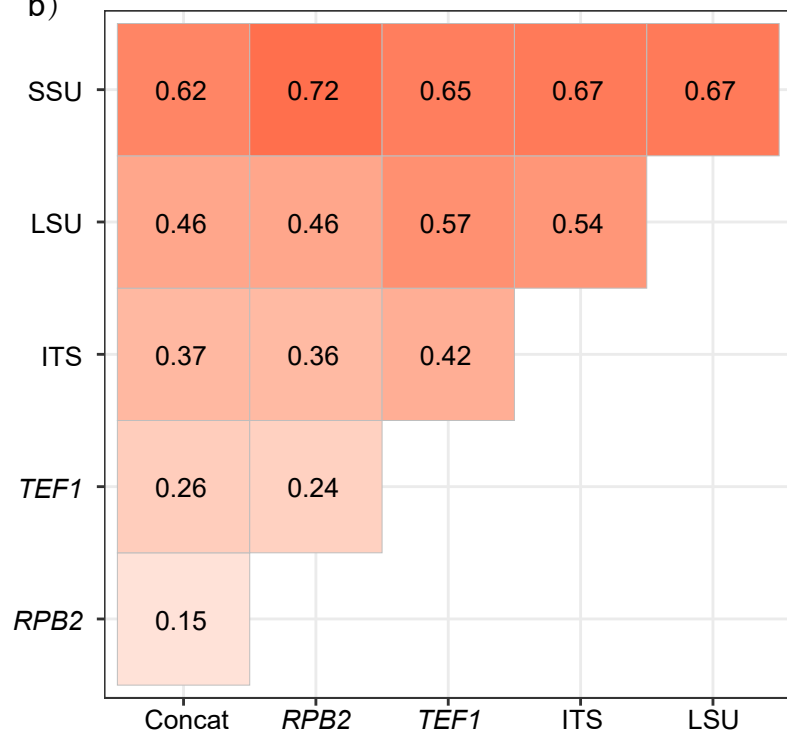

Distance

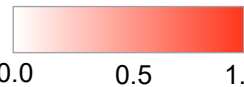

Supplement: Supplementary file 1 [file jof-08-00712-s001.zip › Figure S2.pdf]

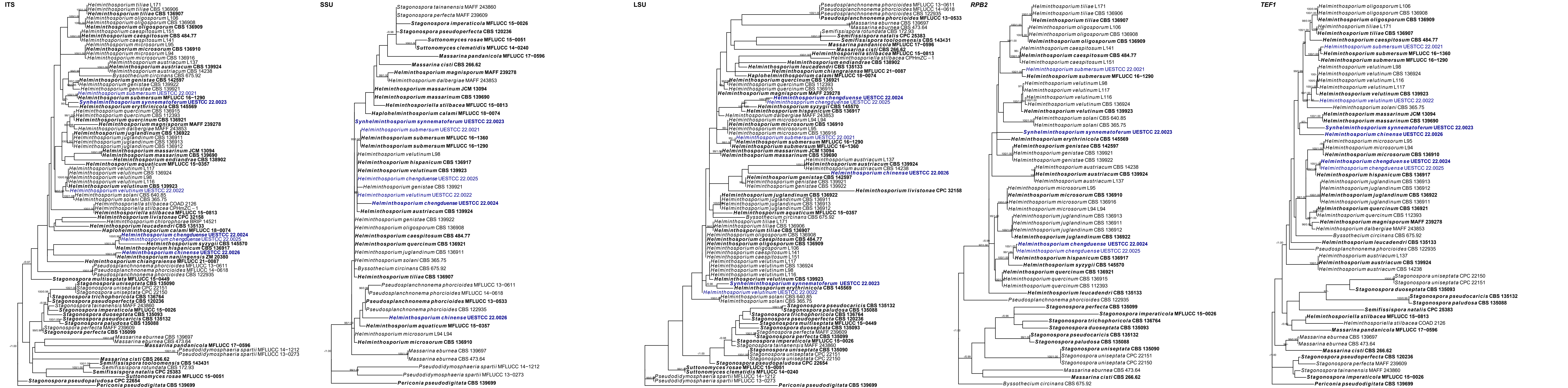

Supplement: Supplementary file 1 [file jof-08-00712-s001.zip › Figure S1.pdf]
